# Do Cancer Genetics Impact Treatment Decision Making? Immunotherapy and Beyond in the Management of Advanced and Metastatic Urothelial Carcinoma

**DOI:** 10.3390/curroncol30080536

**Published:** 2023-08-04

**Authors:** Gavin Hui, Dimitrios Stefanoudakis, Yuliya Zektser, Dayna Jill Isaacs, Christopher Hannigan, Allan J. Pantuck, Alexandra Drakaki

**Affiliations:** 1 Department of Medicine, Division of Hematology and Oncology, David Geffen School of Medicine, Los Angeles, CA 90095, USA; 2 School of Medicine, National & Kapodistrian University of Athens, 15772 Athens, Greece; 3 Department of Medicine, David Geffen School of Medicine, Los Angeles, CA 90095, USA; 4 Department of Urology, David Geffen School of Medicine, Los Angeles, CA 90095, USA

**Keywords:** urothelial carcinoma, immunotherapy, cancer, genetics

## Abstract

Bladder cancer is one of the most commonly diagnosed genitourinary malignancies. For many years, the primary treatment for metastatic urothelial cancer (mUC) was predicated on the use of platinum-based chemotherapy. More recently, immune checkpoint inhibitors (ICIs) were approved by regulatory agencies such as the US FDA for use in both the first- and second-line settings. This review outlines the approved ICIs for mUC in the second-line setting and as an alternative to chemotherapy in the first-line setting, as well as the novel agents that have also been incorporated into the treatment of this malignancy. Single-agent ICIs are often used in second-line settings in mUC, and there are three drugs currently approved for those who progress after receiving platinum-based chemotherapy. In the first-line setting, the preferred treatment regimen remains cisplatin-based chemotherapy. However, single-agent ICI can be an alternative first-line treatment for those who are not candidates for cisplatin-based therapy. There are also clinical trials adding ICIs to chemotherapy as combination regimens. However, treatment for mUC has now expanded even beyond immunotherapy. Newer targeted agents such as erdafitinib, a fibroblast growth factor receptor inhibitor, and two antibody–drug conjugates, enfortumab vedotin and sacituzumab govitecan, have been recently approved. As new drug agents are discovered, it will be important to assess both the treatment outcomes as well as the effects on patients’ quality of life. Furthermore, integrating genetic and molecular information can help guide treatment decisions as next-generation sequencing is more commonly acquired during the evaluation of newly diagnosed patients with advanced and metastatic cancer.

## 1. Introduction

Urothelial cancer is a polychronotopic malignancy that is known to arise from epithelial cells lining the entire length of the urinary tract, including the renal pelvis, ureter, bladder, and urethra. Among urothelial cancers, the bladder is the most common site of involvement. In the United States, there were an estimated 83,730 new cases of bladder cancer and 17,200 deaths from this disease in 2021 [1]. In comparison, in 1978, there were an estimated 30,000 new cases and 9900 deaths. For many years, the treatment for metastatic urothelial cancer (mUC) was based on the use of multi-agent, platinum-based chemotherapy. However, new immunotherapy drugs and increased utilization of molecular analysis have given us hope for additional, and more rationally chosen, treatment options.

Advances in bioinformatics have further characterized and subtyped urothelial cancers according to their genetic and protein expression profiles, which have far-reaching implications for both therapy and prognosis. Like breast cancers, bladder cancers have been categorized into two major subtypes via mRNA expression profiling: luminal and basal [2]. Basal subtypes are characterized by p63 activation, squamous differentiation, and more aggressive disease. They have a higher burden of immune infiltration and express higher levels of the immune checkpoint ligands programmed death-ligand 1 (PD-L1) and CTLA-4 as compared to luminal tumors [3]. Luminal mUC, on the other hand, is driven by PPARg and estrogen receptor transcription. They are enriched with activating FGFR3 mutations and thus yield the potential for FGFR inhibitor sensitivity. There is a third subtype of mUC, however, that expresses luminal biomarkers but is also distinguished by a wild-type p53 gene expression signature, referred to as “p53-like”. Metastatic UCs with this expression pattern have been shown to be resistant to neoadjuvant cisplatin-based combination chemotherapy [4]. Focusing on genetic profiling, there have been efforts to characterize the DNA damage and repair genes that affect the response to cisplatin. For example, ERCC2 encodes a nucleotide excision repair helicase, and it was found that mutations in this gene confer cisplatin sensitivity and may be used to predict the response [5,6]. Given that genotypic and molecular phenotypes affect prognosis and possibly therapeutic choice, research efforts are being directed toward validating immunohistochemical classifiers for standardized use.

## 2. Chemotherapy

Until relatively recently, there were limited systemic treatments for metastatic bladder cancer, and patients faced a grim prognosis. A study in the 1980s on patients with mUC showed that the mean duration of survival was only 13 months from the time of diagnosis until death [7]. The common sites of metastasis identified in this study included the lung, bones, lymph nodes, and liver. This suggested that mUC required more effective approaches to systemic treatment.

Effective chemotherapy for mUC was developed in the 1980s. In 1985, the MVAC (methotrexate, vinblastine, doxorubicin, and cisplatin) regimen was discovered to be effective in a cohort of 25 patients with mUC [8]. Complete clinical remission was seen in half of this cohort, and the preliminary results of this initial study paved the way for the use of MVAC chemotherapy in treating advanced mUC. With its accelerated use throughout the 1990s, MVAC became the standard approach for treating patients with advanced urothelial cancer. However, the toxicities and short duration of response of the MVAC regimen limited its utility. Over time, newer treatment combinations were tested and compared favorably to the MVAC regimen. For example, another regimen using gemcitabine and cisplatin (GC) was found to have similar survival outcomes with greater tolerability. These chemotherapies showed high initial response rates, with a median survival of 15 months [9,10].

The common toxicities of cisplatin-based treatments include kidney damage, hearing loss, and peripheral neuropathy. Therefore, patients with poor performance status, renal dysfunction, and other comorbidities such as congestive heart failure may not be optimal candidates to receive cisplatin. Patients deemed ineligible for cisplatin-based chemotherapy are generally then considered for carboplatin-based combination chemotherapy because of its greater tolerability and decreased toxicity profile.

In the early 2000s, there were no standard treatments for patients who were refractory to first-line platinum chemotherapy. Historically, other types of chemotherapies were studied to treat mUC in the second-line setting. These treatments included taxanes, such as paclitaxel and docetaxel, and anti-metabolites, such as pemetrexed. A single-group study from Canada showed that nab-paclitaxel was well tolerated in advanced UC patients who had been treated with a platinum-based regimen already. The ORR was 27.7% (17.3–44.4), and the most common side effects were fatigue, alopecia, and neuropathy [11]. A newer study in 2020 found that paclitaxel had similar efficacy to nab-paclitaxel, and the ORR was encouraging enough to be a reasonable option for patients with platinum-refractory mUC [12].

In the mid-2000s, a phase II clinical study involving 47 patients treated with pemetrexed with locally advanced or metastatic mUC demonstrated 3 (6.4%) complete responses and 10 (21.3%) partial responses, suggesting that single-agent pemetrexed may be an option as a second-line treatment for advanced mUC [13]. These studies supported alternative options for second-line treatment of mUC. However, with the introduction of checkpoint inhibitor-based immunotherapy, there have been many more drugs approved for the treatment of mUC.

## 3. Immune Checkpoint Inhibitor Genomics

Tumor cells evade the immune system by expressing immune checkpoint proteins [14,15,16]. ICIs are monoclonal antibodies that enhance anti-tumor, T-cell-mediated activity by blocking either the cytotoxic T-lymphocyte-associated antigen 4 (CTLA-4)/B7 or the programmed cell death protein-1 (PD-1)/programmed death ligand-1 (PD-L1) immune checkpoint pathways, thereby activating the immune system [17]. The CTLA-4 and PD-1 receptors on T-cells normally bind to the B7 and PD-L1 receptors on antigen-presenting cells, respectively, to regulate the immune system.

The majority of evidence demonstrates the importance of PD-L1 as a biomarker for urothelial cell cancer despite the numerous variations in quantifying this protein, including the use of different diagnostic assays, scoring systems, and cut-off values. Increased PD-L1 tumor expression generally correlates with a more favorable response to treatment with ICIs [18]. Some studies suggest a threshold of 5% or greater PD-L1 tumor expression to treat with ICIs [19]. However, this correlation is imperfect, as low levels of PD-L1 expression in tumor cells have also been associated with positive clinical responses.

ICIs are clinically effective for reasons that are two-fold. First, they target tumor cells that intrinsically express PD-L1 proteins, blocking the binding of the ligand to the associated checkpoint receptor. Second, the high mutational burden of urothelial cell cancer activates various pathways associated with the expression of immune checkpoint proteins and the formation of neoantigens [20]. Urothelial cell cancer has the third highest mutation rate among all cancers, following only lung cancer and melanoma [21]. The Cancer Genome Atlas project obtained mean and median somatic mutation rates of 7.7 and 5.5 per megabase, respectively [22]. This high mutational burden increases the probability of developing mutations along pathways such as the mammalian target of rapamycin (mTOR) and p53/RB pathways. The mTOR pathway regulates transcription and protein synthesis to promote cancer cell proliferation. The key genes include FGFR2, PIK3C3, FGFR4, FGFR1, FGF3, AKT1, mTOR, and RPTOR, whose missense mutations have been associated with increased PD-L1 expression [23]. Moreover, the tumor suppressor gene, p53, has been shown to increase transcription and expression of PD-1 in tumor cells through acetylation at K120/164 [24]. Lastly, through clonal expansion of T-cells, ICIs have been shown to enhance the immune system’s ability to detect and eliminate neoantigens in tumor cells arising as a result of somatic mutations that are capable of binding with major histocompatibility complexes for presentation to T-cells [25,26].

## 4. Immune Checkpoint Inhibitor Approvals and Indications

With the approval of several ICIs, the second-line treatment of patients with metastatic UC has changed dramatically [27]. There are currently three drugs, avelumab, pembrolizumab, and nivolumab, approved in the US for the second-line treatment of mUC patients who have progressed after receiving platinum-based therapy.

Avelumab, a PD-L1 inhibitor, was granted accelerated FDA approval on 9 May 2017, and it is used to treat patients with locally advanced or mUC in the second-line setting. A phase I study with a total of 161 patients who received avelumab and had at least 6 months of follow-up demonstrated an ORR of 17% [28].

Pembrolizumab, a second PD-1 inhibitor, was approved based on evidence of improved OS in refractory mUC patients. The KEYNOTE-045 trial compared patients on pembrolizumab versus chemotherapy, and the OS was 10.1 months vs. 7.3 months, respectively, at a median follow-up of 28 months [29]. Based on KEYNOTE-045, pembrolizumab received regular approval from the FDA to treat patients with locally advanced or mUC in the second-line setting [30].

The third agent to be approved for second-line mUC treatment is nivolumab, another PD-1 inhibitor. In both phase I and phase II studies, nivolumab achieved durable clinical responses in patients who progressed on platinum therapy. The CheckMate275 trial was the landmark trial that led to the approval of nivolumab as a therapeutic option for treating patients in the second-line setting. The overall ORR was 19.6% in 270 patients treated with nivolumab. Furthermore, significant objective responses were observed in both cohorts of patients with PD-L1 expression of 1% or less as well as those with PD-L1 expression of 1% or greater. This showed a treatment response irrespective of the levels of PD-L1 expression [31]. On 2 February 2017, nivolumab was granted accelerated approval from the FDA to treat locally advanced or mUC patients in this second-line setting [32].

While avelumab, pembrolizumab, and nivolumab are second-line options, there were previously two other drugs approved for the same indications, making a total of five ICIs that were available for use in treating advanced UC. However, with updated study results suggesting no benefit over chemotherapy, the FDA approvals for durvalumab and atezolizumab, both PD-L1 inhibitors, were subsequently withdrawn. On 1 May 2017, durvalumab was granted accelerated approval by the US FDA in the second-line therapy setting [33]. This approval was based on a trial of 182 patients with locally advanced or mUC who had disease progression after initial platinum-based therapy. The ORR in these 182 patients was 26.3% in those with higher PD-L1 scores based on the VENTANA PD-L1 (SP263) assay. However, a phase III trial (DANUBE) with a larger cohort of 1032 patients did not find significantly improved survival benefits in those with durvalumab versus the chemotherapy group, and this included the high PD-L1 expression group [34]. Based on the results of this phase III trial, FDA approval for durvalumab in the second-line setting was voluntarily withdrawn in the US. Similarly, atezolizumab was also voluntarily withdrawn after initial accelerated approval by the US FDA for second-line treatment in locally advanced or mUC. Initial phase I and II clinical trials suggested an improvement in the OS for locally advanced or mUC, with higher response in those with higher PD-L1 expression [35]. However, the preliminary analysis of the phase III IMvigor 211 trial of 931 patients found that the OS was similar between atezolizumab versus chemotherapy, thus prompting the withdrawal of its approval as a second-line therapy. More recently, IMvigor 211 was completed and the updated study results showed a 24-month OS rate of 23% with atezolizumab and 13% with chemotherapy during a median of 33 months of follow-up [36]. This suggests there may be a significant benefit of atezolizumab over chemotherapy in the second-line setting after all.

## 5. First-Line Immunotherapy

The preferred first-line systemic therapy for mUC remains cisplatin-based regimens. However, not all patients are good candidates for cisplatin. As noted above, cisplatin is known to cause renal dysfunction, hearing loss, and peripheral neuropathy. Systemic ICI-based immunotherapy is now considered an alternative first-line option for patients not eligible for platinum-based chemotherapy (cisplatin- or carboplatin-based), regardless of the level of PD-L1 expression.

Atezolizumab was evaluated as a first-line treatment in a single-arm multicenter study. It included 119 patients in 47 academic medical centers. These patients had locally advanced or mUC and they were previously untreated or ineligible for cisplatin therapy. The ORR was 23% at the 17.2-month median follow-up [37]. There were responses across all the PD-L1 expression subgroups. Based on this initial study, atezolizumab was granted accelerated approval in 2017 for patients who are not eligible for cisplatin-based chemotherapy. However, this approval was revised based on additional phase III study results. Currently, atezolizumab is used as a first-line therapy in those who express PD-L1 and are ineligible for cisplatin, or in anyone who cannot receive any platinum-based chemotherapy.

Pembrolizumab was also studied as another first-line option in patients who were ineligible for cisplatin, including those who were elderly and had poor prognostic factors, such as medical comorbidities or poor performance scores. KEYNOTE-361 found that the OS was higher in ICI plus chemotherapy versus chemotherapy alone; however, it was not statistically significant [38]. Therefore, pembrolizumab cannot be added to standard chemotherapy in the first-line setting. Currently, it is used as a single agent in first-line therapy in only platinum-ineligible patients.

Another role of ICI-based immunotherapy is maintenance therapy, which is used in patients who achieve objective responses after platinum-based chemotherapy. In this setting, pembrolizumab and avelumab have been studied; however, avelumab is the only FDA-approved maintenance therapy. Avelumab has been found to prolong OS compared to supportive care alone in patients who achieve objective response after platinum-based chemotherapy. The median OS was 21.4 months in those receiving avelumab versus 14.3 months in those receiving supportive care [39].

## 6. ICI Combination Therapy in the First-Line Setting

Therapy with ICIs has changed the landscape of first-line and second-line mUC therapy with single-agent treatments. More recently, however, there have been studies to determine if the addition of immunotherapy to chemotherapy as a combination regimen could lead to better outcomes. Below are several trials studying an immunotherapy combination regimen in the first-line setting.

The KEYNOTE-361 open-label MK-3475-361/KEYNOTE-361 phase III clinical trial enrolled 1010 participants with locally advanced or mUC to compare the efficacy and safety of pembrolizumab with or without chemotherapy versus chemotherapy alone. The chemotherapy entailed a combination of gemcitabine with platinum therapy (cisplatin or carboplatin). The trial had three arms, pembrolizumab plus chemotherapy, pembrolizumab, and chemotherapy. The median progression-free survival times for the pembrolizumab plus chemotherapy versus chemotherapy were 8.3 months and 7.1 months, and the median overall survival times were 17 months and 14.3 months, respectively. This trial concluded that pembrolizumab plus chemotherapy versus chemotherapy alone did not significantly improve PFS or OS.

Another trial was IMvigorr130, a phase III trial that enrolled 1200 patients to compare atezolizumab combined with platinum-based chemotherapy versus chemotherapy with placebo in the first-line setting. Patients were randomized into three groups, atezolizumab with platinum-based chemotherapy, atezolizumab monotherapy, and placebo plus platinum-based chemotherapy. The median PFS in the intention-to-treat population was 8.2 months (6.5–8.3) in the combination therapy group versus 6.3 months (6.2–7) in the placebo with chemotherapy arm [40]. This study suggests the combination of atezolizumab with platinum-based chemotherapy could be a potential first-line mUC treatment option.

The DANUBE open-label phase III clinical trial enrolled 1032 participants with locally advanced or mUC to evaluate the efficacy and safety of durvalumab and durvalumab in combination with tremelimumab, a CTLA-4 inhibitor. The chemotherapy entailed gemcitabine plus cisplatin or gemcitabine plus carboplatin. Randomization was stratified by cisplatin eligibility, PD-L1 status, and the presence or absence of liver metastases, lung metastases, or both. The primary endpoint was overall survival. The trial had three arms: durvalumab, durvalumab plus tremelimumab, and chemotherapy alone. In the high PD-L1 population, the median overall survival time was 14.4 months in the durvalumab group and 12.1 months in the chemotherapy group. In the intention-to-treat population, the median survival time was 15.1 months in the durvalumab plus tremelimumab combination group and 12.1 months in the chemotherapy group. This trial found that durvalumab and tremelimumab combination therapy was not superior to chemotherapy alone.

At the time of writing this manuscript, ongoing clinical trials are evaluating the efficacies of ICIs, and these trials include NILE and PEMBRO/EV. The NILE (NCT03682068) open-label, ongoing phase III clinical trial, which enrolled 1434 participants with unresectable locally advanced urothelial cancer or mUC, aims to evaluate the efficacy and safety of durvalumab as well as durvalumab in combination with tremelimumab compared with chemotherapy. The PEMBRO/EV open-label trial is an ongoing phase III clinical trial that enrolled 760 participants with untreated locally advanced or mUC. This trial aims to evaluate enfortumab vedotin (EV), a targeted therapy comprising an anti-nectin-4 antibody–drug conjugate (ADC) and pembrolizumab versus standard-of-care chemotherapy. This trial has three arms: enfortumab vedotin with pembrolizumab, gemcitabine with cisplatin or carboplatin, and enfortumab vedotin with pembrolizumab and cisplatin or carboplatin.

The data thus far are not compelling for the addition of ICIs to first-line chemotherapy. Pending their anticipated completion, the NILE and PEMBRO/EV trials may provide more clarity for the first-line setting, including whether combining ICIs is beneficial. Currently, patients ineligible for cisplatin-based chemotherapy may benefit from treatment with atezolizumab or pembrolizumab. Avelumab remains the only FDA-approved ICI for maintenance therapy.

## 7. ICI Combinations in the Second-Line Setting

The combination of ICIs for second-line treatment appears promising in an early-phase trial, although further study is needed. The two trials to date are the Checkmate 032 and MORPHEUS studies. The Checkmate 032 trial was a basket phase I/II clinical research trial for patients across six different indications, including mUC [41]. The arms included nivolumab monotherapy every 2 weeks and 2 dose variations of the nivolumab and ipilimumab combination followed by nivolumab monotherapy. The greatest objective response was in the group receiving nivolumab 3 mg/kg plus ipilimumab 1 mg/kg every 3 weeks for 4 doses, followed by nivolumab monotherapy 3 mg/kg every 2 weeks. This arm had an ORR of 38.0%, the highest among the three randomized groups. Checkmate 032 suggested the potential benefit of using nivolumab and ipilimumab combination therapy in mUC.

The MORPHEUS trial is an ongoing phase Ib/II master protocol for patients with cisplatin-ineligible muscle-invasive bladder cancer (MIBC) and locally advanced or mUC who have progressed with platinum-based regimens [42]. The mUC cohort is divided into one control arm (atezolizumab) and eight experimental arms tested thus far, with seven in combination with atezolizumab (enfortumab vedotin, niraparib, Hu5F9-G4, sacituzumab govitecan, tocilizumab, tiragolumab, and RO7122290) and the last with RO7122290 monotherapy. This study will randomize approximately 735 patients based on their PD-L1 expression levels. The primary efficacy endpoint is the overall response rate in the mUC cohort and the pathological complete response (pCR) in the MIBC cohort. The safety endpoint is to evaluate the toxicity of immunotherapy combinations across all the experimental arms. This study is still open to enrollment but several of the phase Ib cohorts have closed for preliminary data analysis.

## 8. Antibody–Drug Conjugates: Enfortumab Vedotin (EV) and Sacituzumab Govitecan (SG)

While ICIs have provided new treatment options for mUC, antibody–drug conjugates (ADCs) represent a new class of promising therapy being tested in patients with locally advanced or mUC. ADCs utilize the specificity of antibody–antigen interaction to deliver small-molecule anticancer drugs directly to the targeted cells. The potential benefits include systemic antibody-specific targeting combined with local cytotoxic antitumor activity.

One such ADC was discussed earlier in this paper as part of the PEMBRO/EV and MORPHEUS trial. Enfortumab vedotin (EV) targets nectin-4, a tumor-associated antigen that is highly expressed in mUC, and it delivers monomethyl auristatin E (MMAE), a synthetic, highly-toxic antineoplastic agent. EV first showed efficacy in the phase II EV-201 trial for patients with locally advanced or mUC previously treated with platinum chemotherapy and anti-PD-1/L1 therapy. Out of 125 patients, the confirmed ORR was 44%, with a median time to response of 1.84 months and a median duration of response of 7.6 months. The phase III study of patients in the same population compared with the third-line investigator choice chemotherapy showed a superior median OS, with 12.88 months in the EV arm and 8.97 months in the chemotherapy arm. The phase II and phase III studies showed similar safety profiles, and the most commonly reported AEs in the EV cohorts were alopecia 49% and 45.3%, peripheral neuropathy 40% and 33.8%, fatigue 50% and 31.1%, and decreased appetite 44% and 30.7%, respectively.

Sacituzumab govitecan (SG) is a Trop-2-directed ADC that was given accelerated FDA approval in April 2021 for locally advanced or mUC and triple-negative breast cancer. This ADC targets a transmembrane glycoprotein (Trop-2). The drug has a coupled active metabolite, SN-38. SN-38 is a small molecule anti-tumor agent and the active metabolite of irinotecan. Initially tested in the phase I IMMU-132 study, SG showed efficacy with an RR of 50% (*n* = 6) and a tolerable safety profile in the platinum-resistant mUC setting [43]. The follow-up phase II TROPHY-U-01 study in patients post-platinum therapy demonstrated more statistically significant results (*n* = 113), with ORR seen in 27.4%, 5.3% CR, and 22.1% PR. It should also be noted that three of the PRs (*n* = 10) were patients previously treated with EV [26]. The most commonly reported AEs were diarrhea 65%, nausea 60%, fatigue 52%, alopecia 47%, neutropenia 46%, and decreased appetite 36%.

## 9. New Targeted Agents

For those with fibroblast growth factor receptor (FGFR) 2 or 3 genetic alterations, erdafitinib, an FGFR inhibitor, is an option. Erdafitinib is FDA-approved for patients with locally advanced or mUC with a susceptible genetic alteration of FGFR2 or FGFR3 after previous treatments [44]. Erdafitinib demonstrated efficacy in a nonrandomized phase II trial with 101 patients. The majority of the patients in this trial had progressed on at least one prior chemotherapy regimen. The objective response rate was 40% of 101 patients [45]. At a median follow-up of 24 months, the median progression-free survival was 6 months and the median overall survival was 11 months. Erdafitinib is a newer targeted therapy; however, there are no direct comparisons of erdafitinib versus chemotherapy or immunotherapy. There are also no direct comparisons of erdafitinib, EV, and SG. Therefore, any of the three can be used in those with FGFR mutations as a later-line treatment. Newer agents targeting the FGFR pathway are in development. Furthermore, clinical trials, such as NCT04963153, are evaluating the combination of erdafitinib with EV. 

## 10. Conclusions

Our paper provides an overview of the treatment therapies for mUC, starting from the initial chemotherapy regimen era to the present day. Over time, immunotherapy, notably PD-1 and PD-L1 ICIs, has been incorporated into the first- and second-line treatment settings. Table 1 provides a summary of the clinical trials. The options for first-line treatment of mUC include atezolizumab and pembrolizumab for patients who are not candidates for cisplatin-based therapy. The second-line FDA-approved treatment alternatives include avelumab, pembrolizumab, and nivolumab. Avelumab remains the only FDA-approved ICI for maintenance therapy. Ongoing studies are exploring the benefits of concurrently administering immunotherapy and chemotherapy, Thus far, only IMVIGOR130 has shown potential benefit, although is not standard practice yet, and we are still awaiting final data from the NILE and CHECKMATE 901 trials. ADCs have also provided additional options for patients who are refractory to platinum-based chemotherapy and/or immunotherapy. Lastly, later-stage treatment with erdafitinib, an FGFR inhibitor, can be used to target tumors with activating fibroblast growth factor receptor mutations. Further studies, however, are still necessary to assess FGFR inhibitors in combination with other agents.

## Figures and Tables

**Table 1:** Clinical Trials for Urothelial Carcinoma.

Clinical Trial	Phase	Chemotherapy Type	Treatment Arms	Number of Patients	Disease Severity	Median Overall Survival Time (Hazard Ratio, 95% Confidence Interval, *p*-Value)	Median Progression Free Survival Time (Hazard Ratio, 95% Confidence Interval, *p*-Value)	Outcome	Status
IMvigor 211	3	First-line	Atezolizumab + chemotherapy vs. chemotherapy	931	Locally advanced or metastatic urothelial cancer	11.1 vs. 10.6 months (HR 0.87, 95% CI 0.63–1.21, *p* = 0.41)	N/A; not an outcome	Not significant	Active, not recruiting
IMvigor 130	3	First-line	Atezolizumab + chemotherapy vs. chemotherapy + placebo	1200	Locally advanced or metastatic urothelial cancer	16.0 months vs. 13.4 months (HR 0.83, 95% CI 0.69–1.00, *p* = 0.027, α = 0.007)	8.2 months vs. 6.3 months (HR 0.82, 95% CI 0.70–0.96, *p* = 0.007)	Significant for improved PFS	Active, not recruiting
MK-3475-361/KEYNOTE-361	3	First-line	Pembrolizumab + standard chemotherapy vs. pembrolizumab vs. standard chemotherapy	1010	Locally advanced or metastatic urothelial cancer	17.0 vs. 14.3 months (HR 0.86, 95% CI 0.72–1.02, *p* = 0.0407, α = 0.0142)	8.3 months vs. 7.1 months (HR 0.78, 95% CI 0.65–0.93, *p* = 0.0033, α = 0.0019)	Not significant	Active, not recruiting
NCT02603432	3	Maintenance	Avelumab + supportive care vs. supportive care	700	Locally advanced or metastatic urothelial cancer who did not have disease progression with first-line chemotherapy	21.4 vs. 14.3 months (HR 0.69, 95% CI 0.56–0.86, *p* = 0.001)	3.7 months vs. 2.0 months (HR 0.62, 95% CI 0.52–0.75)	Significant for improved OS and improved PFS	Active, not recruiting
DANUBE	3	First-line	Durvalumab vs. durvalumab + tremelimumab vs. chemotherapy	1032	Locally advanced or metastatic urothelial cancer	14.4 months (durvalumab alone) vs. 12.1 months (chemotherapy alone) (HR 0.89, 95% CI 0.71–1.11, *p* = 0.30)	2.3 months vs. 3.7 months vs. 6.7 months (statistics not reported)	Not significant	Active, not recruiting
NILE	3	First-Line	Durvalumab + Chemotherapy and Durvalumab + Tremelimumab Chemotherapy vs. Chemotherapy alone	1292	Unresectable Locally Advanced or Metastatic Urothelial Cancer	No results posted yet	No results posted yet	No results posted yet	Recruiting
PEMBRO/EV	3	First-line	Enfortumab Vedotin and Pembrolizumab vs. Chemotherapy	860	Untreated Locally Advanced or Metastatic Urothelial Cancer	Not reached	12.3 months (95% CI: 8.0)	No results posted yet	Recruiting
CheckMate-032	1/2	Second line with Urothelial	Nivolumab vs Nivolumab + Ipilimumab	1131 (78 with UC)	Advanced or Metastatic Solid Tumors: 6 tumor types—triple-negative breast cancer, gastric cancer, pancreatic adenocarcinoma, small cell lung cancer, bladder cancer, and ovarian cancer	Urothelial: 9.9 months (95% CI, 7.3 to 21.1 months) in the NIVO3 arm, 7.4 months (95% CI, 5.6 to 11.0 months) in the NIVO3 + IPI1 arm, and 15.3 months (95% CI, 10.1 to 27.6 months) in the NIVO1 + IPI3 arm	Urothelial: 2.8 months (95% CI, 1.5 to 5.3 months) in NIVO3, 2.6 months (95% CI, 1.4 to 3.9 months) in NIVO3 + IPI1, and 4.9 months (95% CI, 2.7 to 6.6 months) in NIVO3 + IPI1	N/A	Active, not recruiting
MORPHEUS	1b/2	Different lines of treatment	Multiple Immunotherapy-Based Treatments and Combinations (Atezolizumab, Enfortumab Vedotin, Niraparib, Hu5F9-G4, Tiragolumab, Sacituzumab Govitecan, Tocilizumab, Cisplatin, Gemcitabine)	645	GI Cancer, Urothelial Carcinoma, Melanoma	No results posted yet	No results posted yet	No results posted yet	Recruiting
EV-201	2	Second-line	Enfortumab Vedotin	219	Locally advanced or metastatic urothelial carcinoma patients who were previously treated with ICIs	14.7 months (95% CI 10.51–18.2)	5.8 months (95% CI 5.03–8.28)	N/A	Active, not recruiting
TROPHY-U-01	2	Third-Line	Sacituzumab Govitecan	321	Metastatic Urothelial Carcinoma Progressing After Platinum-Based Chemotherapy and Checkpoint Inhibitors	5.4 months (95% CI, 3.5 to 7.2 months)	10.9 months (95% CI, 9.0 to 13.8 months)	Preliminary Data	Recruiting
CHECKMATE 901	3	First-Line	Nivolumab + Ipilimumab or Chemotherapy vs. Chemotherapy Alone	1307	Untreated Inoperable or Metastatic Urothelial Cancer	No results posted yet	No results posted yet	No results posted yet	Recruiting

## References

[1] American Cancer Society Key Statistics for Bladder Cancer. https://www.cancer.org/cancer/bladder-cancer/about/key-statistics.html.

[2] Guo C.C., Bondaruk J., Yao H., Wang Z., Zhang L., Lee S., Lee J.-G., Cogdell D., Zhang M., Yang G. (2020). Assessment of Luminal and Basal Phenotypes in Bladder Cancer. Sci. Rep..

[3] Robertson A.G., Kim J., Al-Ahmadie H., Bellmunt J., Guo G., Cherniack A.D., Hinoue T., Laird P.W., Hoadley K.A., Akbani R. (2018). Comprehensive Molecular Characterization of Muscle-Invasive Bladder Cancer. Cell.

[4] Choi W., Porten S., Kim S., Willis D., Plimack E.R., Hoffman-Censits J., Roth B., Cheng T., Tran M., Lee I.-L. (2014). Identification of Distinct Basal and Luminal Subtypes of Muscle-Invasive Bladder Cancer with Different Sensitivities to Frontline Chemotherapy. Cancer Cell.

[5] Mouw K.W. (2017). DNA Repair Pathway Alterations in Bladder Cancer. Cancers.

[6] Iacovino M.L., Miceli C.C., De Felice M., Barone B., Pompella L., Chiancone F., Di Zazzo E., Tirino G., Della Corte C.M., Imbimbo C. (2022). Novel Therapeutic Opportunities in Neoadjuvant Setting in Urothelial Cancers: A New Horizon Opened by Molecular Classification and Immune Checkpoint Inhibitors. Int. J. Mol. Sci..

[7] Babaian R.J., Johnson D.E., Llamas L., Ayala A.G. (1980). Metastases from transitional cell carcinoma of urinary bladder. Urology.

[8] Sternberg C.N., Yagoda A., Scher H.I., Watson R.C., Ahmed T., Weiselberg L.R., Geller N., Hollander P.S., Herr H.W., Sogani P.C. (1985). Preliminary results of M-VAC (methotrexate, vinblastine, doxorubicin and cisplatin) for transitional cell carcinoma of the urothelium. J. Urol..

[9] von der Maase H., Hansen S.W., Roberts J.T., Dogliotti L., Oliver T., Moore M.J., Bodrogi I., Albers P., Knuth A., Lippert C.M. (2000). Gemcitabine and Cisplatin Versus Methotrexate, Vinblastine, Doxorubicin, and Cisplatin in Advanced or Metastatic Bladder Cancer: Results of a Large, Randomized, Multinational, Multicenter, Phase III Study. J. Clin. Oncol..

[10] von der Maase H., Sengelov L., Roberts J.T., Ricci S., Dogliotti L., Oliver T., Moore M.J., Zimmermann A., Arning M. (2005). Long-term survival results of a randomized trial comparing gemcitabine plus cisplatin, with methotrexate, vinblastine, doxorubicin, plus cisplatin in patients with bladder cancer. J. Clin. Oncol..

[11] Nanoparticle albumin-bound paclitaxel for second-line treatment of metastatic urothelial carcinoma: A single group, multicentre, phase 2 study–PubMed. https://pubmed.ncbi.nlm.nih.gov/23706985/.

[12] Sridhar S.S., Blais N., Tran B., Reaume M.N., North S.A., Stockler M.R., Chi K.N., Fleshner N.E., Liu G., Robinson J.W. (2020). Efficacy and Safety of nab-Paclitaxel vs Paclitaxel on Survival in Patients with Platinum-Refractory Metastatic Urothelial Cancer: The Canadian Cancer Trials Group BL.12 Randomized Clinical Trial. JAMA Oncol..

[13] Sweeney C.J., Roth B.J., Kabbinavar F.F., Vaughn D.J., Arning M., Curiel R.E., Obasaju C.K., Wang Y., Nicol S.J., Kaufman D.S. (2006). Phase II study of pemetrexed for second-line treatment of transitional cell cancer of the urothelium. J. Clin. Oncol..

[14] Pardoll D.M. (2012). The blockade of immune checkpoints in cancer immunotherapy. Nat. Rev. Cancer..

[15] Chen D.S., Mellman I. (2013). Oncology meets immunology: The cancer-immunity cycle. Immunity.

[16] Buchbinder E.I., Desai A. (2016). CTLA-4 and PD-1 Pathways: Similarities, Differences, and Implications of Their Inhibition. Am. J. Clin. Oncol..

[17] Barone B., Calogero A., Scafuri L., Ferro M., Lucarelli G., Di Zazzo E., Sicignano E., Falcone A., Romano L., De Luca L. (2022). Immune Checkpoint Inhibitors as a Neoadjuvant/Adjuvant Treatment of Muscle-Invasive Bladder Cancer: A Systematic Review. Cancers.

[18] Powles T., Walker J., Andrew Williams J., Bellmunt J. (2020). The evolving role of PD-L1 testing in patients with metastatic urothelial carcinoma. Cancer Treat. Rev..

[19] Huang J., Teng X. (2020). Expression of PD-L1 for predicting response to immune checkpoint inhibitors in metastatic urothelial carcinoma: A systematic review and meta-analysis. Curr. Oncol..

[20] Cheng W., Fu D., Xu F., Zhang Z. (2018). Unwrapping the genomic characteristics of urothelial bladder cancer and successes with immune checkpoint blockade therapy. Oncogenesis.

[21] Kim J., Akbani R., Creighton C.J., Lerner S.P., Weinstein J.N., Getz G., Kwiatkowski D.J. (2015). Invasive Bladder Cancer: Genomic Insights and Therapeutic Promise. Clin. Cancer Res..

[22] The Cancer Genome Atlas Research Network (2014). Comprehensive molecular characterization of urothelial bladder carcinoma. Nature.

[23] Cheng L., Wang Y., Qiu L., Chang Y., Lu H., Liu C., Zhang B., Zhou Y., Bai H., Xiong L. (2022). mTOR pathway gene mutations predict response to immune checkpoint inhibitors in multiple cancers. J. Transl. Med..

[24] Cao Z., Kon N., Liu Y., Xu W., Wen J., Yao H., Zhang M., Wu Z., Yan X., Zhu W.-G. (2021). An unexpected role for p53 in regulating cancer cell-intrinsic PD-1 by acetylation. Sci. Adv..

[25] Lee C.H., Yelensky R., Jooss K., Chan T.A. (2018). Update on Tumor Neoantigens and Their Utility: Why It Is Good to Be Different. Trends Immunol..

[26] Schumacher T.N., Schreiber R.D. (2015). Neoantigens in cancer immunotherapy. Science.

[27] Bellmunt J., Théodore C., Demkov T., Komyakov B., Sengelov L., Daugaard G., Caty A., Carles J., Jagiello-Gruszfeld A., Karyakin O. (2009). Phase III trial of vinflunine plus best supportive care compared with best supportive care alone after a platinum-containing regimen in patients with advanced transitional cell carcinoma of the urothelial tract. J. Clin. Oncol..

[28] Patel M.R., Ellerton J., Infante J.R., Agrawal M., Gordon M., Aljumaily R., Britten C.D., Dirix L., Lee K.-W., Taylor M. (2018). Avelumab in metastatic urothelial carcinoma after platinum failure (JAVELIN Solid Tumor): Pooled results from two expansion cohorts of an open-label, phase 1 trial. Lancet Oncol..

[29] Pembrolizumab as Second-Line Therapy for Advanced Urothelial Carcinoma|NEJM. https://www.nejm.org/doi/10.1056/NEJMoa1613683?url_ver=Z39.88-2003&rfr_id=ori:rid:crossref.org&rfr_dat=cr_pub%3dwww.ncbi.nlm.nih.go.

[30] Research C for DE and Pembrolizumab (Keytruda): Advanced or Metastatic Urothelial Carcinoma. FDA. https://www.fda.gov/drugs/resources-information-approved-drugs/pembrolizumab-keytruda-advanced-or-metastatic-urothelial-carcinoma.

[31] Sharma P., Retz M., Siefker-Radtke A., Baron A., Necchi A., Bedke J., Plimack E.R., Vaena D., Grimm M.-O., Bracarda S. (2017). Nivolumab in metastatic urothelial carcinoma after platinum therapy (CheckMate 275): A multicentre, single-arm, phase 2 trial. Lancet Oncol..

[32] Research C for DE and Nivolumab for Treatment of Urothelial Carcinoma. FDA. https://www.fda.gov/drugs/resources-information-approved-drugs/nivolumab-treatment-urothelial-carcinoma.

[33] Research C for DE and Durvalumab (Imfinzi). FDA. https://www.fda.gov/drugs/resources-information-approved-drugs/durvalumab-imfinzi.

[34] Powles T., van der Heijden M.S., Castellano D., Galsky M.D., Loriot Y., Petrylak D.P., Ogawa O., Park S.H., Lee J.-L., De Giorgi U. (2020). Durvalumab alone and durvalumab plus tremelimumab versus chemotherapy in previously untreated patients with unresectable, locally advanced or metastatic urothelial carcinoma (DANUBE): A randomised, open-label, multicentre, phase 3 trial. Lancet Oncol..

[35] Rosenberg J.E., Hoffman-Censits J., Powles T., van der Heijden M.S., Balar A.V., Necchi A., Dawson N., O’Donnell P.H., Balmanoukian A., Loriot Y. (2016). Atezolizumab in patients with locally advanced and metastatic urothelial carcinoma who have progressed following treatment with platinum-based chemotherapy: A single-arm, multicentre, phase 2 trial. Lancet.

[36] van der Heijden M.S., Loriot Y., Durán I., Ravaud A., Retz M., Vogelzang N.J., Nelson B., Wang J., Shen X., Powles T. (2021). Atezolizumab Versus Chemotherapy in Patients with Platinum-treated Locally Advanced or Metastatic Urothelial Carcinoma: A Long-term Overall Survival and Safety Update from the Phase 3 IMvigor211 Clinical Trial. Eur. Urol..

[37] Balar A.V., Galsky M.D., Rosenberg J.E., Powles T., Petrylak D.P., Bellmunt J., Loriot Y., Necchi A., Hoffman-Censits J., Perez-Gracia J.L. (2017). Atezolizumab as first-line treatment in cisplatin-ineligible patients with locally advanced and metastatic urothelial carcinoma: A single-arm, multicentre, phase 2 trial. Lancet.

[38] Powles T., Csőszi T., Özgüroğlu M., Matsubara N., Géczi L., Cheng S.Y.-S., Fradet Y., Oudard S., Vulsteke C., Barrera R.M. (2021). Pembrolizumab alone or combined with chemotherapy versus chemotherapy as first-line therapy for advanced urothelial carcinoma (KEYNOTE-361): A randomised, open-label, phase 3 trial. Lancet Oncol..

[39] Powles T., Park S.H., Voog E., Caserta C., Valderrama B.P., Gurney H., Kalofonos H., Radulović S., Demey W., Ullén A. (2020). Avelumab Maintenance Therapy for Advanced or Metastatic Urothelial Carcinoma. N. Engl. J. Med..

[40] Galsky M.D., Arija J.Á.A., Bamias A., Davis I.D., De Santis M., Kikuchi E., Garcia-Del-Muro X., De Giorgi U., Mencinger M., Izumi K. (2020). Atezolizumab with or without chemotherapy in metastatic urothelial cancer (IMvigor130): A multicentre, randomised, placebo-controlled phase 3 trial. Lancet.

[41] Sharma P., Siefker-Radtke A., De Braud F., Basso U., Calvo E., Bono P., Morse M.A., Ascierto P.A., Lopez-Martin J., Brossart P. (2019). Nivolumab Alone and With Ipilimumab in Previously Treated Metastatic Urothelial Carcinoma: CheckMate 032 Nivolumab 1 mg/kg Plus Ipilimumab 3 mg/kg Expansion Cohort Results. J. Clin. Oncol..

[42] Drakaki A., Kalebasty A.R., Lee J.-L., Martin-Liberal J., Kim M., Shin S.J., Shi J., Mariathasan S., Ci B., Degaonkar V. (2020). Phase Ib/II umbrella trial to evaluate the safety and efficacy of multiple 2L cancer immunotherapy (CIT) combinations in advanced/metastatic urothelial carcinoma (mUC): MORPHEUS-mUC. JCO.

[43] Faltas B., Goldenberg D.M., Ocean A.J., Govindan S.V., Wilhelm F., Sharkey R.M., Hajdenberg J., Hodes G., Nanus D.M., Tagawa S.T. (2016). Sacituzumab Govitecan, a Novel Antibody--Drug Conjugate, in Patients with Metastatic Platinum-Resistant Urothelial Carcinoma. Clin. Genitourin. Cancer.

[44] Loriot Y., Necchi A., Park S.H., Garcia-Donas J., Huddart R., Burgess E., Fleming M., Rezazadeh A., Mellado B., Varlamov S. (2019). Erdafitinib in Locally Advanced or Metastatic Urothelial Carcinoma. N. Engl. J. Med..

[45] Siefker-Radtke A., Necchi A., Park S.H., García-Donas J., A Huddart R., Burgess E.F., Fleming M.T., Kalebasty A.R., Mellado B., Varlamov S. (2022). Efficacy and safety of erdafitinib in patients with locally advanced or metastatic urothelial carcinoma: Long-term follow-up of a phase 2 study. Lancet Oncol..

